# Ethyl (*Z*)-3-(4-methyl­anilino)-2-[(4-methyl­phen­yl)carbamo­yl]prop-2-enoate

**DOI:** 10.1107/S1600536812043723

**Published:** 2012-10-27

**Authors:** Arun M. Islor, B. Garudachari, Thomas Gerber, Eric Hosten, Richard Betz

**Affiliations:** a National Institute of Technology-Karnataka, Department of Chemistry, Medicinal Chemistry Laboratory, Surathkal, Mangalore 575 025, India; b Nelson Mandela Metropolitan University, Summerstrand Campus, Department of Chemistry, University Way, Summerstrand, PO Box 77000, Port Elizabeth, 6031, South Africa

## Abstract

The title compound, C_20_H_22_N_2_O_3_, is a secondary amine featuring an amide and an ester functionality in connection with a Michael system. The conformation about the C=C bond is *E*. Intra­molecular N—H⋯O hydrogen bonds occur. In the crystal, C—H⋯O contacts connect the mol­ecules into chains along the *b-*axis direction.

## Related literature

For general information about the synthetic and industrial importance of aniline and its derivatives, see: Berry & Royd (1984 ▶); Garudachari *et al.* (2012 ▶); Sridharan *et al.* (2006 ▶); Kasthuri *et al.* (2008 ▶). For graph-set analysis of hydrogen bonds, see: Etter *et al.* (1990 ▶); Bernstein *et al.* (1995 ▶).
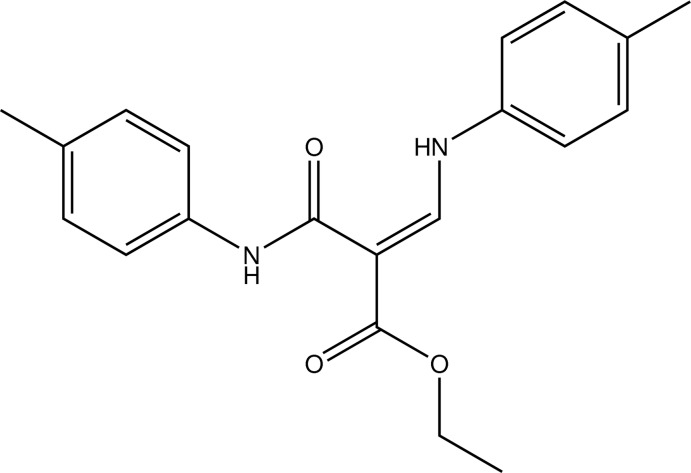


## Experimental

### 

#### Crystal data

C_20_H_22_N_2_O_3_
*M* *_r_* = 338.40
Monoclinic, 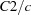

*a* = 18.8170 (4) Å
*b* = 11.9752 (3) Å
*c* = 15.6043 (4) Å
β = 91.470 (1)°
*V* = 3515.07 (15) Å^3^
*Z* = 8
Mo *K*α radiation
μ = 0.09 mm^−1^
*T* = 200 K
0.42 × 0.26 × 0.19 mm

#### Data collection

Bruker APEXII CCD diffractometer
Absorption correction: multi-scan (*SADABS*; Bruker, 2008 ▶) *T*_min_ = 0.965, *T*_max_ = 0.984
16569 measured reflections
4353 independent reflections
3411 reflections with *I* > 2σ(*I*)
*R*_int_ = 0.022

#### Refinement

*R*[*F* ^2^ > 2σ(*F* ^2^)] = 0.051
*wR*(*F* ^2^) = 0.147
*S* = 1.05
4353 reflections
237 parameters
H atoms treated by a mixture of independent and constrained refinement
Δρ_max_ = 0.56 e Å^−3^
Δρ_min_ = −0.22 e Å^−3^

### 

Data collection: *APEX2* (Bruker, 2010 ▶); cell refinement: *SAINT* (Bruker, 2010 ▶); data reduction: *SAINT*; program(s) used to solve structure: *SHELXS97* (Sheldrick, 2008 ▶); program(s) used to refine structure: *SHELXL97* (Sheldrick, 2008 ▶); molecular graphics: *ORTEP-3* (Farrugia, 1997 ▶) and *Mercury* (Macrae *et al.*, 2008 ▶); software used to prepare material for publication: *SHELXL97* and *PLATON* (Spek, 2009 ▶).

## Supplementary Material

Crystal structure: contains datablock(s) I, global. DOI: 10.1107/S1600536812043723/zq2185sup1.cif

Supplementary material file. DOI: 10.1107/S1600536812043723/zq2185Isup2.cdx

Structure factors: contains datablock(s) I. DOI: 10.1107/S1600536812043723/zq2185Isup3.hkl

Supplementary material file. DOI: 10.1107/S1600536812043723/zq2185Isup4.cml

Additional supplementary materials:  crystallographic information; 3D view; checkCIF report

## Figures and Tables

**Table 1:** Hydrogen-bond geometry (Å, °)

*D*—H⋯*A*	*D*—H	H⋯*A*	*D*⋯*A*	*D*—H⋯*A*
C23—H23⋯O1^i^	0.95	2.68	3.620 (2)	170
C25—H25⋯O2^ii^	0.95	2.70	3.4685 (19)	139
N1—H1⋯O1	0.97 (2)	1.85 (2)	2.6383 (17)	135.9 (18)
N2—H2⋯O2	0.88 (2)	1.92 (2)	2.6713 (18)	143.0 (19)

Symmetry codes: (i) 
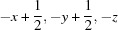
; (ii) 
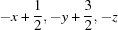
.

**Fig. 1.**
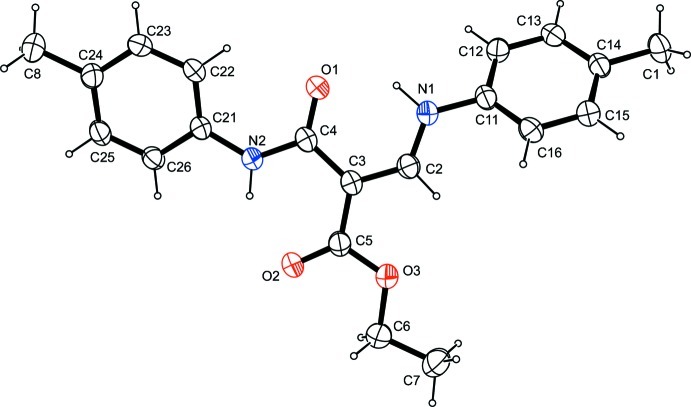
The molecular structure of the title compound, with atom labels and anisotropic displacement ellipsoids (drawn at 50% probability level).

**Fig. 2.**
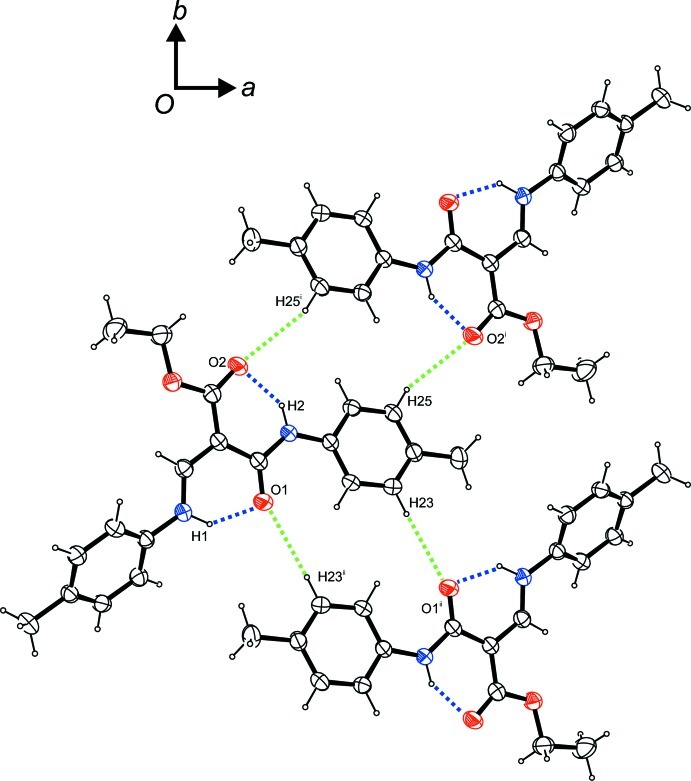
Intermolecular contacts, viewed along [0 0 - 1]. Intermolecular C–H···O contacts are depicted with green dashed lines, intramolecular N–H···O hydrogen bonds are depicted with blue dashed lines. Symmetry operators: (i) -x + 1/2, -y + 3/2, -z; (ii) -x + 1/2, -y + 1/2, -z.

**Fig. 3.**
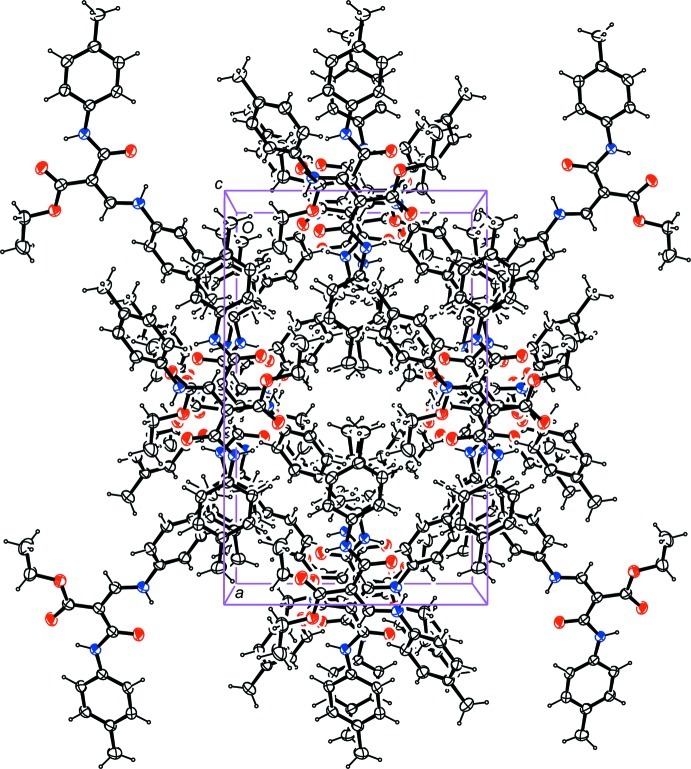
Molecular packing of the title compound, viewed along [0 0 - 1] (anisotropic displacement ellipsoids drawn at 50% probability level).

## supplementary crystallographic information

### Comment

The study of aniline derivatives is important due to the presence of amines in natural products and nucleic acids (Berry & Royd, 1984). Aniline compounds find widespread applications in the field of synthetic chemistry such as the synthesis of quinolines and indoles (Garudachari *et al.*, 2012; Sridharan *et al.*, 2006). Aniline derivatives are also widely used in many industries such as in the production of dyes and agrochemicals (Kasthuri *et al.*, 2008). Keeping in mind the importance of aniline derivatives, the title compound was synthesized to study its crystal structure.

The molecule can – simultaneously – be regarded as a secondary amide, an enamine, an ester as well as featuring a Michael system. The C=C bond is (*E*) configured. The least-squares planes defined by the respective carbon atoms of the phenyl rings intersect at an angle of 49.57 (8) °. The central part of the molecule, including the ethyl group, is essentially planar (r.m.s. of the least-squares plane defined by all the non-hydrogen atoms of the respective part of this molecule = 0.0569 Å) with the oxygen atom of the ethoxy group deviating most from this plane (0.095 (1) Å) (Fig. 1).

In the crystal, intramolecular N–H···O bonds involving all secondary amine groups and double bonded oxygen atoms are observed. In addition, intermolecular C–H···O contacts whose range falls slightly below the sum of van-der-Waals radii of the atoms participating are present. The latter contacts are supported by hydrogen atoms on the phenyl group that is bonded to the amide-type nitrogen atom and exclusively have ketonic oxygen atoms as acceptors. In terms of graph-set analysis (Etter *et al.*, 1990; Bernstein *et al.*, 1995), the descriptor for these contacts is *S*(6)*S*(6)*R*^2^_2_(14)*R*^2^_2_(18) on the unary level. Metrical parameters as well as information about the symmetry of these contacts are summarized in Table 1. In total, the molecules are connected to chains along the crystallographic *b* axis. The shortest intercentroid distance between two aromatic systems was measured at 4.5754 (9) Å and is observed between the two different aromatic moieties in neighbouring molecules (Fig 2).

The packing of the title compound in the crystal structure is shown in Figure 3.

### Experimental

A mixture of diethyl-{[(4-methylphenyl)amino]methylidene}propanedioate (1.0 g, 0.0036 mol) and 4-methylaniline (0.19 g, 0.0018 mol) in dowtherm (10 ml) was stirred at 150 °C for 2 h. The reaction mixture was then cooled to 25 °C and stirred in *n*-hexane (20 ml) for 10 min. The solid product obtained was filtered, dried and further purified by column chromatography using petrol ether and ethyl acetate (*v*:*v* = 5:5) as the eluent to get a white solid. Crystals were grown by slow evaporation of a dilute ethanol solution at room temperature, yield: 0.52 g (42.6%).

### Refinement

Carbon-bound H atoms were placed in calculated positions (C—H = 0.95 Å for aromatic and vinylic carbon atoms, C—H = 0.99 Å for the methylene group, and C—H = 0.98 Å for the methyl groups) and were included in the refinement in the riding model approximation, with *U*_iso_(H) set to 1.2 or 1.5*U*_eq_(C). The H atoms of the methyl groups were allowed to rotate with a fixed angle around the C—C bond to best fit the experimental electron density [HFIX 137 in the *SHELX* program suite (Sheldrick, 2008), with *U*_iso_(H) set to 1.5*U*_eq_(C)]. Both nitrogen-bound H atoms were located on a difference Fourier map and refined freely.

### Crystal data

**Table:**

C_20_H_22_N_2_O_3_	*F*(000) = 1440
*M**_r_* = 338.40	*D*_x_ = 1.279 Mg m^−^^3^
Monoclinic, *C*2/*c*	Melting point = 438–440 K
Hall symbol: -C 2yc	Mo *K*α radiation, λ = 0.71073 Å
*a* = 18.8170 (4) Å	Cell parameters from 7492 reflections
*b* = 11.9752 (3) Å	θ = 2.4–28.3°
*c* = 15.6043 (4) Å	µ = 0.09 mm^−^^1^
β = 91.470 (1)°	*T* = 200 K
*V* = 3515.07 (15) Å^3^	Cubic, white
*Z* = 8	0.42 × 0.26 × 0.19 mm

### Data collection

**Table:**

Bruker APEXII CCD diffractometer	4353 independent reflections
Radiation source: fine-focus sealed tube	3411 reflections with *I* > 2σ(*I*)
Graphite monochromator	*R*_int_ = 0.022
φ and ω scans	θ_max_ = 28.3°, θ_min_ = 2.0°
Absorption correction: multi-scan (*SADABS*; Bruker, 2008)	*h* = −18→25
*T*_min_ = 0.965, *T*_max_ = 0.984	*k* = −15→11
16569 measured reflections	*l* = −20→20

### Refinement

**Table:**

Refinement on *F*^2^	Primary atom site location: structure-invariant direct methods
Least-squares matrix: full	Secondary atom site location: difference Fourier map
*R*[*F*^2^ > 2σ(*F*^2^)] = 0.051	Hydrogen site location: inferred from neighbouring sites
*wR*(*F*^2^) = 0.147	H atoms treated by a mixture of independent and constrained refinement
*S* = 1.05	*w* = 1/[σ^2^(*F*_o_^2^) + (0.0721*P*)^2^ + 2.7716*P*] where *P* = (*F*_o_^2^ + 2*F*_c_^2^)/3
4353 reflections	(Δ/σ)_max_ < 0.001
237 parameters	Δρ_max_ = 0.56 e Å^−^^3^
0 restraints	Δρ_min_ = −0.22 e Å^−^^3^

### Fractional atomic coordinates and isotropic or equivalent isotropic displacement parameters (Å^2^)

**Table:**

	*x*	*y*	*z*	*U*_iso_*/*U*_eq_	
O1	0.10013 (6)	0.36356 (9)	0.05742 (8)	0.0390 (3)	
O2	0.06034 (7)	0.70809 (10)	0.07895 (9)	0.0432 (3)	
O3	−0.03953 (6)	0.66632 (10)	0.14650 (8)	0.0396 (3)	
N1	−0.02327 (7)	0.33906 (11)	0.13174 (9)	0.0328 (3)	
N2	0.14011 (7)	0.53822 (11)	0.02606 (9)	0.0311 (3)	
C1	−0.25933 (9)	0.06696 (16)	0.22528 (12)	0.0427 (4)	
H1A	−0.3026	0.0976	0.1980	0.064*	
H1B	−0.2645	0.0658	0.2876	0.064*	
H1C	−0.2516	−0.0092	0.2046	0.064*	
C2	−0.02178 (8)	0.44917 (13)	0.13478 (10)	0.0312 (3)	
H2A	−0.0606	0.4854	0.1608	0.037*	
C3	0.03155 (8)	0.51666 (13)	0.10328 (9)	0.0297 (3)	
C4	0.09288 (8)	0.46684 (13)	0.06104 (9)	0.0302 (3)	
C5	0.02073 (8)	0.63730 (14)	0.10765 (10)	0.0326 (3)	
C6	−0.05772 (10)	0.78362 (15)	0.14399 (13)	0.0469 (4)	
H6A	−0.0622	0.8099	0.0839	0.056*	
H6B	−0.0205	0.8283	0.1741	0.056*	
C7	−0.12693 (12)	0.79542 (19)	0.18756 (17)	0.0635 (6)	
H7A	−0.1402	0.8745	0.1898	0.095*	
H7B	−0.1224	0.7658	0.2460	0.095*	
H7C	−0.1637	0.7537	0.1556	0.095*	
C8	0.41549 (10)	0.47429 (18)	−0.11452 (14)	0.0508 (5)	
H8A	0.4139	0.4875	−0.1765	0.076*	
H8B	0.4322	0.3981	−0.1029	0.076*	
H8C	0.4481	0.5278	−0.0868	0.076*	
C11	−0.08114 (8)	0.27242 (13)	0.15835 (10)	0.0309 (3)	
C12	−0.09618 (8)	0.17563 (14)	0.11323 (10)	0.0341 (3)	
H12	−0.0674	0.1541	0.0669	0.041*	
C13	−0.15332 (9)	0.11012 (13)	0.13590 (10)	0.0334 (3)	
H13	−0.1632	0.0436	0.1046	0.040*	
C14	−0.19666 (8)	0.13884 (13)	0.20320 (10)	0.0318 (3)	
C15	−0.18046 (9)	0.23622 (14)	0.24851 (10)	0.0345 (3)	
H15	−0.2091	0.2575	0.2951	0.041*	
C16	−0.12299 (9)	0.30289 (13)	0.22665 (10)	0.0339 (3)	
H16	−0.1125	0.3689	0.2583	0.041*	
C21	0.20602 (8)	0.51483 (13)	−0.01130 (10)	0.0291 (3)	
C22	0.24017 (9)	0.41154 (13)	−0.00733 (11)	0.0348 (4)	
H22	0.2177	0.3494	0.0184	0.042*	
C23	0.30724 (9)	0.40025 (13)	−0.04125 (11)	0.0371 (4)	
H23	0.3301	0.3296	−0.0382	0.044*	
C24	0.34214 (8)	0.48883 (14)	−0.07960 (11)	0.0355 (4)	
C25	0.30692 (9)	0.59088 (14)	−0.08402 (10)	0.0350 (4)	
H25	0.3293	0.6528	−0.1102	0.042*	
C26	0.23978 (8)	0.60390 (13)	−0.05100 (10)	0.0315 (3)	
H26	0.2165	0.6741	−0.0554	0.038*	
H1	0.0165 (11)	0.3084 (18)	0.1007 (13)	0.051 (6)*	
H2	0.1286 (11)	0.6090 (19)	0.0307 (13)	0.046 (6)*	

### Atomic displacement parameters (Å^2^)

**Table:**

	*U*^11^	*U*^22^	*U*^33^	*U*^12^	*U*^13^	*U*^23^
O1	0.0374 (6)	0.0282 (6)	0.0518 (7)	−0.0027 (5)	0.0111 (5)	0.0020 (5)
O2	0.0395 (6)	0.0313 (6)	0.0595 (8)	−0.0067 (5)	0.0168 (6)	0.0000 (5)
O3	0.0365 (6)	0.0332 (6)	0.0498 (7)	−0.0006 (5)	0.0139 (5)	0.0026 (5)
N1	0.0278 (6)	0.0320 (7)	0.0389 (7)	−0.0026 (5)	0.0048 (5)	0.0011 (5)
N2	0.0281 (6)	0.0269 (7)	0.0384 (7)	−0.0020 (5)	0.0039 (5)	0.0021 (5)
C1	0.0386 (9)	0.0414 (10)	0.0482 (10)	−0.0135 (7)	0.0049 (7)	0.0033 (8)
C2	0.0305 (7)	0.0325 (8)	0.0304 (7)	−0.0024 (6)	−0.0008 (6)	0.0011 (6)
C3	0.0286 (7)	0.0308 (8)	0.0297 (7)	−0.0047 (6)	−0.0001 (6)	0.0017 (6)
C4	0.0293 (7)	0.0311 (8)	0.0300 (7)	−0.0058 (6)	−0.0017 (6)	0.0026 (6)
C5	0.0309 (7)	0.0343 (8)	0.0329 (8)	−0.0039 (6)	0.0028 (6)	0.0001 (6)
C6	0.0472 (10)	0.0346 (9)	0.0599 (12)	0.0025 (8)	0.0159 (9)	0.0037 (8)
C7	0.0595 (13)	0.0477 (12)	0.0848 (16)	0.0138 (10)	0.0327 (12)	0.0098 (11)
C8	0.0335 (9)	0.0503 (11)	0.0694 (13)	−0.0037 (8)	0.0150 (9)	−0.0061 (9)
C11	0.0273 (7)	0.0308 (8)	0.0346 (8)	−0.0032 (6)	0.0000 (6)	0.0066 (6)
C12	0.0322 (8)	0.0349 (8)	0.0355 (8)	−0.0002 (6)	0.0042 (6)	0.0022 (6)
C13	0.0355 (8)	0.0277 (7)	0.0368 (8)	−0.0023 (6)	−0.0009 (6)	0.0017 (6)
C14	0.0285 (7)	0.0305 (8)	0.0365 (8)	−0.0034 (6)	−0.0004 (6)	0.0068 (6)
C15	0.0337 (8)	0.0346 (8)	0.0353 (8)	−0.0024 (6)	0.0034 (6)	0.0018 (6)
C16	0.0355 (8)	0.0311 (8)	0.0350 (8)	−0.0059 (6)	−0.0015 (6)	−0.0004 (6)
C21	0.0270 (7)	0.0288 (7)	0.0315 (7)	−0.0043 (6)	0.0003 (6)	0.0002 (6)
C22	0.0328 (8)	0.0256 (7)	0.0462 (9)	−0.0055 (6)	0.0041 (7)	0.0021 (6)
C23	0.0343 (8)	0.0261 (7)	0.0509 (10)	−0.0009 (6)	0.0024 (7)	−0.0028 (7)
C24	0.0291 (7)	0.0356 (8)	0.0419 (9)	−0.0043 (6)	0.0034 (6)	−0.0046 (7)
C25	0.0335 (8)	0.0325 (8)	0.0390 (8)	−0.0080 (6)	0.0043 (6)	0.0031 (6)
C26	0.0314 (8)	0.0274 (7)	0.0357 (8)	−0.0021 (6)	−0.0002 (6)	0.0037 (6)

### Geometric parameters (Å, º)

**Table:**

O1—C4	1.2458 (19)	C8—C24	1.507 (2)
O2—C5	1.2214 (19)	C8—H8A	0.9800
O3—C5	1.3451 (19)	C8—H8B	0.9800
O3—C6	1.446 (2)	C8—H8C	0.9800
N1—C2	1.320 (2)	C11—C12	1.382 (2)
N1—C11	1.4207 (19)	C11—C16	1.390 (2)
N1—H1	0.97 (2)	C12—C13	1.384 (2)
N2—C4	1.3576 (19)	C12—H12	0.9500
N2—C21	1.412 (2)	C13—C14	1.389 (2)
N2—H2	0.88 (2)	C13—H13	0.9500
C1—C14	1.507 (2)	C14—C15	1.393 (2)
C1—H1A	0.9800	C15—C16	1.394 (2)
C1—H1B	0.9800	C15—H15	0.9500
C1—H1C	0.9800	C16—H16	0.9500
C2—C3	1.388 (2)	C21—C22	1.395 (2)
C2—H2A	0.9500	C21—C26	1.395 (2)
C3—C5	1.461 (2)	C22—C23	1.388 (2)
C3—C4	1.470 (2)	C22—H22	0.9500
C6—C7	1.491 (3)	C23—C24	1.391 (2)
C6—H6A	0.9900	C23—H23	0.9500
C6—H6B	0.9900	C24—C25	1.391 (2)
C7—H7A	0.9800	C25—C26	1.385 (2)
C7—H7B	0.9800	C25—H25	0.9500
C7—H7C	0.9800	C26—H26	0.9500
			
C5—O3—C6	116.13 (13)	C24—C8—H8C	109.5
C2—N1—C11	124.50 (14)	H8A—C8—H8C	109.5
C2—N1—H1	112.3 (13)	H8B—C8—H8C	109.5
C11—N1—H1	122.6 (13)	C12—C11—C16	119.89 (14)
C4—N2—C21	129.21 (14)	C12—C11—N1	118.09 (14)
C4—N2—H2	114.2 (14)	C16—C11—N1	122.02 (14)
C21—N2—H2	116.5 (13)	C11—C12—C13	119.70 (15)
C14—C1—H1A	109.5	C11—C12—H12	120.1
C14—C1—H1B	109.5	C13—C12—H12	120.1
H1A—C1—H1B	109.5	C12—C13—C14	121.85 (15)
C14—C1—H1C	109.5	C12—C13—H13	119.1
H1A—C1—H1C	109.5	C14—C13—H13	119.1
H1B—C1—H1C	109.5	C13—C14—C15	117.76 (14)
N1—C2—C3	125.72 (15)	C13—C14—C1	120.62 (15)
N1—C2—H2A	117.1	C15—C14—C1	121.62 (15)
C3—C2—H2A	117.1	C14—C15—C16	121.08 (15)
C2—C3—C5	117.15 (14)	C14—C15—H15	119.5
C2—C3—C4	120.36 (14)	C16—C15—H15	119.5
C5—C3—C4	122.29 (13)	C11—C16—C15	119.72 (15)
O1—C4—N2	122.23 (14)	C11—C16—H16	120.1
O1—C4—C3	120.75 (13)	C15—C16—H16	120.1
N2—C4—C3	117.02 (14)	C22—C21—C26	118.90 (14)
O2—C5—O3	121.02 (15)	C22—C21—N2	124.49 (14)
O2—C5—C3	125.61 (15)	C26—C21—N2	116.53 (14)
O3—C5—C3	113.37 (13)	C23—C22—C21	119.48 (14)
O3—C6—C7	106.77 (15)	C23—C22—H22	120.3
O3—C6—H6A	110.4	C21—C22—H22	120.3
C7—C6—H6A	110.4	C22—C23—C24	122.27 (15)
O3—C6—H6B	110.4	C22—C23—H23	118.9
C7—C6—H6B	110.4	C24—C23—H23	118.9
H6A—C6—H6B	108.6	C23—C24—C25	117.49 (15)
C6—C7—H7A	109.5	C23—C24—C8	120.95 (16)
C6—C7—H7B	109.5	C25—C24—C8	121.56 (16)
H7A—C7—H7B	109.5	C26—C25—C24	121.22 (15)
C6—C7—H7C	109.5	C26—C25—H25	119.4
H7A—C7—H7C	109.5	C24—C25—H25	119.4
H7B—C7—H7C	109.5	C25—C26—C21	120.62 (15)
C24—C8—H8A	109.5	C25—C26—H26	119.7
C24—C8—H8B	109.5	C21—C26—H26	119.7
H8A—C8—H8B	109.5		
			
C11—N1—C2—C3	−174.78 (14)	C11—C12—C13—C14	0.1 (2)
N1—C2—C3—C5	175.86 (15)	C12—C13—C14—C15	−0.6 (2)
N1—C2—C3—C4	0.9 (2)	C12—C13—C14—C1	178.83 (15)
C21—N2—C4—O1	−6.6 (2)	C13—C14—C15—C16	0.4 (2)
C21—N2—C4—C3	173.92 (14)	C1—C14—C15—C16	−179.03 (15)
C2—C3—C4—O1	−4.3 (2)	C12—C11—C16—C15	−0.8 (2)
C5—C3—C4—O1	−179.08 (14)	N1—C11—C16—C15	178.07 (14)
C2—C3—C4—N2	175.10 (14)	C14—C15—C16—C11	0.3 (2)
C5—C3—C4—N2	0.4 (2)	C4—N2—C21—C22	−10.2 (3)
C6—O3—C5—O2	6.2 (2)	C4—N2—C21—C26	172.94 (15)
C6—O3—C5—C3	−172.92 (15)	C26—C21—C22—C23	1.3 (2)
C2—C3—C5—O2	−175.98 (15)	N2—C21—C22—C23	−175.49 (15)
C4—C3—C5—O2	−1.1 (3)	C21—C22—C23—C24	−0.1 (3)
C2—C3—C5—O3	3.1 (2)	C22—C23—C24—C25	−0.7 (3)
C4—C3—C5—O3	178.03 (13)	C22—C23—C24—C8	178.98 (17)
C5—O3—C6—C7	177.95 (16)	C23—C24—C25—C26	0.4 (2)
C2—N1—C11—C12	144.73 (16)	C8—C24—C25—C26	−179.34 (16)
C2—N1—C11—C16	−34.1 (2)	C24—C25—C26—C21	0.8 (2)
C16—C11—C12—C13	0.6 (2)	C22—C21—C26—C25	−1.7 (2)
N1—C11—C12—C13	−178.31 (14)	N2—C21—C26—C25	175.37 (14)

### Hydrogen-bond geometry (Å, º)

**Table:**

*D*—H···*A*	*D*—H	H···*A*	*D*···*A*	*D*—H···*A*
C23—H23···O1^i^	0.95	2.68	3.620 (2)	170
C25—H25···O2^ii^	0.95	2.70	3.4685 (19)	139
N1—H1···O1	0.97 (2)	1.85 (2)	2.6383 (17)	135.9 (18)
N2—H2···O2	0.88 (2)	1.92 (2)	2.6713 (18)	143.0 (19)

Symmetry codes: (i) −*x*+1/2, −*y*+1/2, −*z*; (ii) −*x*+1/2, −*y*+3/2, −*z*.
